# *tert*-Butyl *N*-(thio­phen-2-yl)carbamate

**DOI:** 10.1107/S160053681302196X

**Published:** 2013-08-14

**Authors:** Gene C. Hsu, Laci M. Singer, David B. Cordes, Michael Findlater

**Affiliations:** a Department of Chemistry & Biochemistry, Texas Tech University, Memorial Circle & Boston, Lubbock, TX 79409, USA

## Abstract

In the title compound, C_9_H_13_NO_2_S, the dihedral angle between the thiophene ring and the carbamate group is 15.79 (14)°. In the crystal structure, intra­molecular C—H⋯O inter­actions in tandem with the *tert*-butyl groups render the packing of adjacent mol­ecules in the [001] direction nearly perpendicular [the angle between adjacent thio­phene rings is 74.83 (7)°]. An inter­molecular N—H⋯O hydrogen bond gives rise to a chain extending along [001]. The crystal studied was found to be a racemic twin.

## Related literature

For the synthesis of the title compound, see: Binder *et al.* (1977 ▶); Kruse *et al.* (1989 ▶). For related structures, see: Arsenyan *et al.* (2008 ▶); Elshaarawy & Janiak (2011 ▶); Low *et al.* (2009 ▶); Hsu *et al.* (2013 ▶).
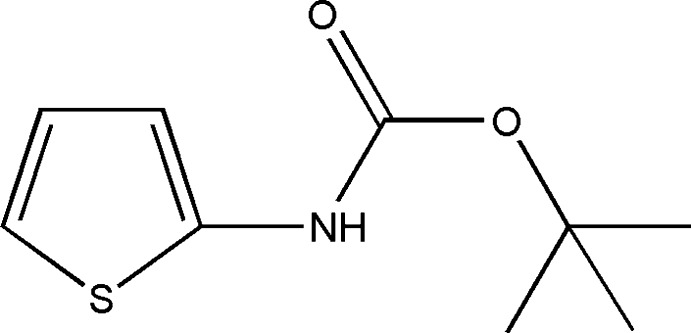


## Experimental

### 

#### Crystal data

C_9_H_13_NO_2_S
*M* *_r_* = 199.26
Orthorhombic, 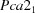

*a* = 11.732 (2) Å
*b* = 8.6513 (17) Å
*c* = 9.879 (2) Å
*V* = 1002.7 (3) Å^3^
*Z* = 4
Mo *K*α radiation
μ = 0.29 mm^−1^
*T* = 153 K
0.20 × 0.10 × 0.08 mm

#### Data collection

Nonius KappaCCD diffractometer
Absorption correction: multi-scan (*DENZO* and *SCALEPACK*; Otwinowski & Minor, 1997 ▶) *T*_min_ = 0.944, *T*_max_ = 0.977
2112 measured reflections
2112 independent reflections
1816 reflections with *I* > 2σ(*I*)

#### Refinement

*R*[*F* ^2^ > 2σ(*F* ^2^)] = 0.034
*wR*(*F* ^2^) = 0.078
*S* = 1.04
2112 reflections
125 parameters
2 restraints
H atoms treated by a mixture of independent and constrained refinement
Δρ_max_ = 0.25 e Å^−3^
Δρ_min_ = −0.19 e Å^−3^
Absolute structure: Flack *x* determined using 703 quotients [(I^+^)−(I^−^)]/[(I^+^)+(I^−^)] (Parsons & Flack, 2004 ▶)
Absolute structure parameter: 0.53 (4)

### 

Data collection: *COLLECT* (Nonius, 1998 ▶); cell refinement: *COLLECT*; data reduction: *DENZO* and *SCALEPACK* (Otwinowski & Minor, 1997 ▶); program(s) used to solve structure: *SIR97* (Altomare *et al.*, 1999 ▶); program(s) used to refine structure: *SHELXL97* (Sheldrick, 2008 ▶); molecular graphics: *SHELXTL* (Sheldrick, 2008 ▶); software used to prepare material for publication: *SHELXTL*, *enCIFer* (Allen *et al.*, 2004 ▶) and *publCIF* (Westrip, 2010 ▶).

## Supplementary Material

Crystal structure: contains datablock(s) global, I. DOI: 10.1107/S160053681302196X/zs2273sup1.cif

Structure factors: contains datablock(s) I. DOI: 10.1107/S160053681302196X/zs2273Isup2.hkl

Supplementary material file. DOI: 10.1107/S160053681302196X/zs2273Isup3.cml

Additional supplementary materials:  crystallographic information; 3D view; checkCIF report

## Figures and Tables

**Table 1:** Hydrogen-bond geometry (Å, °)

*D*—H⋯*A*	*D*—H	H⋯*A*	*D*⋯*A*	*D*—H⋯*A*
N1—H1*N*⋯O1^i^	0.90 (2)	2.04 (2)	2.920 (3)	165 (3)
C7—H7*A*⋯O1	0.98	2.33	2.938 (4)	119
C8—H8*C*⋯O1	0.98	2.55	3.109 (4)	116

Symmetry code: (i) 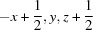.

**Fig. 1.**
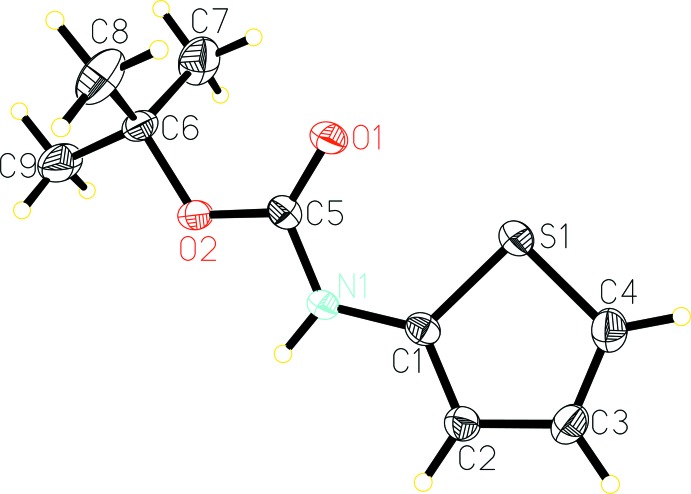
The molecular structure of the title compound with displacement ellipsoids drawn at the 50% probability level.

## supplementary crystallographic information

### 1. Comment

The title compound *tert*-butyl *N*-(thiophene-2-yl)carbamate, C_9_H_13_NO_2_S, (Fig. 1) is a precursor in the synthesis of diimine ligands suitable for metal complex formation. This compound exhibits intramolecular methyl C7—H···O1 and C8—H···O1 interactions [2.938 (4) and 3.109 (4), respectively] in addition to bulky *tert* -butyl groups. These two features in tandem allow the packing in the crystal to be nearly perpendicular [the angle between adjacent thiophene rings = 74.83 (7)°]. An intermolecular N1—H···O1^i^ hydrogen bond (Table 1) gives a one-dimensional chain which extends along [0 0 1]. The compound was synthesized *via* a typical Curtius Rearrangement from thiophene-2-carbonyl azide (Binder *et al.*, 1977; Kruse *et al.*, 1989).

### 2. Experimental

The title compound was prepared by a typical Curtius Rearrangement. Thiophene-2-carbonyl azide (270 mg; 1.77 mmol) was reacted with 1.0 equivalent of *tert*-butyl alcohol (131 mg; 1.77 mmol) and dissolved in 15 ml of toluene. The solution was heated at 100 °C overnight. Excess solvent and *tert*-butyl alcohol was removed *in vacuo*. Crystals suitable for X-ray structure determination were obtained by cooling a toluene solution to -30 °C. ^1^H NMR (400 MHz, chloroform-d): δ=6.9(br, 1H, –NH), 6.79(m, 2H, –CH), 6.5(dd, 1H, –CH), 1.5(s, 9H, tBu).

### 3. Refinement

The NH hydrogen atom was located from the difference-Fourier map and refined isotropically subject to a distance restraint (N—H = 0.98 Å). Carbon-bound H atoms were included in calculated positions (C—H distances are 0.98 Å for methyl H atoms and 0.95 Å for thiophene H atoms) and refined as riding atoms with *U*_iso_(H) = 1.2 *U*_eq_(thiophene H atom) or *U*_iso_(H) = 1.5 *U*_eq_(methyl H atom).

### Crystal data

**Table:**

C_9_H_13_NO_2_S	*D*_x_ = 1.320 Mg m^−^^3^
*M**_r_* = 199.26	Mo *K*α radiation, λ = 0.71073 Å
Orthorhombic, *P**c**a*2_1_	Cell parameters from 1327 reflections
*a* = 11.732 (2) Å	θ = 1.0–27.5°
*b* = 8.6513 (17) Å	µ = 0.29 mm^−^^1^
*c* = 9.879 (2) Å	*T* = 153 K
*V* = 1002.7 (3) Å^3^	Rod, colorless
*Z* = 4	0.20 × 0.10 × 0.08 mm
*F*(000) = 424	

### Data collection

**Table:**

Nonius KappaCCD diffractometer	2112 independent reflections
Radiation source: fine-focus sealed tube	1816 reflections with *I* > 2σ(*I*)
φ and ω scans	θ_max_ = 27.5°, θ_min_ = 2.4°
Absorption correction: multi-scan (*DENZO* and *SCALEPACK*; Otwinowski & Minor, 1997)	*h* = −15→15
*T*_min_ = 0.944, *T*_max_ = 0.977	*k* = −11→11
2112 measured reflections	*l* = −12→12

### Refinement

**Table:**

Refinement on *F*^2^	Secondary atom site location: difference Fourier map
Least-squares matrix: full	Hydrogen site location: mixed
*R*[*F*^2^ > 2σ(*F*^2^)] = 0.034	H atoms treated by a mixture of independent and constrained refinement
*wR*(*F*^2^) = 0.078	*w* = 1/[σ^2^(*F*_o_^2^) + (0.0301*P*)^2^ + 0.2397*P*] where *P* = (*F*_o_^2^ + 2*F*_c_^2^)/3
*S* = 1.04	(Δ/σ)_max_ < 0.001
2112 reflections	Δρ_max_ = 0.25 e Å^−^^3^
125 parameters	Δρ_min_ = −0.19 e Å^−^^3^
2 restraints	Absolute structure: Flack x determined using 703 quotients [(I+)-(I-)]/[(I+)+(I-)] (Parsons & Flack, 2004).
Primary atom site location: structure-invariant direct methods	Absolute structure parameter: 0.53 (4)

### Special details

**Table:**

Geometry. All e.s.d.'s (except the e.s.d. in the dihedral angle between two l.s. planes) are estimated using the full covariance matrix. The cell e.s.d.'s are taken into account individually in the estimation of e.s.d.'s in distances, angles and torsion angles; correlations between e.s.d.'s in cell parameters are only used when they are defined by crystal symmetry. An approximate (isotropic) treatment of cell e.s.d.'s is used for estimating e.s.d.'s involving l.s. planes.

### Fractional atomic coordinates and isotropic or equivalent isotropic displacement parameters (Å^2^)

**Table:**

	*x*	*y*	*z*	*U*_iso_*/*U*_eq_	
S1	0.13622 (6)	0.05380 (7)	0.63359 (7)	0.02745 (19)	
O1	0.29399 (16)	0.2992 (2)	0.61913 (19)	0.0273 (4)	
O2	0.32467 (18)	0.4564 (2)	0.80079 (18)	0.0289 (5)	
N1	0.23352 (19)	0.2367 (2)	0.8305 (2)	0.0224 (5)	
H1N	0.238 (3)	0.264 (4)	0.919 (2)	0.036 (9)*	
C1	0.1713 (2)	0.1041 (3)	0.7987 (3)	0.0209 (5)	
C2	0.1219 (3)	0.0067 (3)	0.8902 (3)	0.0241 (6)	
H2	0.1317	0.0150	0.9854	0.029*	
C3	0.0542 (2)	−0.1082 (3)	0.8269 (3)	0.0289 (6)	
H3	0.0137	−0.1855	0.8754	0.035*	
C4	0.0533 (3)	−0.0964 (3)	0.6907 (3)	0.0306 (7)	
H4	0.0115	−0.1635	0.6329	0.037*	
C5	0.2857 (2)	0.3295 (3)	0.7389 (3)	0.0220 (6)	
C6	0.3798 (2)	0.5829 (3)	0.7233 (3)	0.0250 (6)	
C7	0.3034 (3)	0.6385 (4)	0.6108 (4)	0.0438 (9)	
H7A	0.2928	0.5554	0.5446	0.066*	
H7B	0.3384	0.7280	0.5663	0.066*	
H7C	0.2292	0.6684	0.6483	0.066*	
C8	0.4940 (3)	0.5271 (4)	0.6722 (4)	0.0445 (9)	
H8A	0.5384	0.4856	0.7480	0.067*	
H8B	0.5354	0.6135	0.6310	0.067*	
H8C	0.4823	0.4458	0.6045	0.067*	
C9	0.3945 (3)	0.7083 (3)	0.8284 (4)	0.0459 (9)	
H9A	0.3196	0.7408	0.8617	0.069*	
H9B	0.4337	0.7970	0.7878	0.069*	
H9C	0.4399	0.6685	0.9040	0.069*	

### Atomic displacement parameters (Å^2^)

**Table:**

	*U*^11^	*U*^22^	*U*^33^	*U*^12^	*U*^13^	*U*^23^
S1	0.0369 (4)	0.0261 (3)	0.0194 (3)	−0.0050 (3)	0.0003 (3)	−0.0034 (3)
O1	0.0377 (11)	0.0268 (9)	0.0173 (10)	−0.0043 (8)	0.0014 (9)	−0.0012 (8)
O2	0.0425 (12)	0.0258 (10)	0.0182 (9)	−0.0120 (8)	0.0029 (9)	0.0003 (8)
N1	0.0293 (13)	0.0232 (11)	0.0147 (9)	−0.0051 (9)	0.0006 (10)	−0.0009 (10)
C1	0.0234 (13)	0.0211 (12)	0.0183 (12)	0.0033 (11)	0.0000 (11)	−0.0012 (11)
C2	0.0277 (16)	0.0226 (13)	0.0221 (13)	0.0002 (11)	−0.0017 (11)	0.0006 (11)
C3	0.0300 (16)	0.0227 (14)	0.0340 (15)	−0.0037 (12)	−0.0002 (13)	0.0029 (13)
C4	0.0329 (18)	0.0259 (15)	0.0330 (15)	−0.0030 (12)	−0.0029 (14)	−0.0039 (13)
C5	0.0238 (15)	0.0227 (14)	0.0195 (15)	0.0016 (11)	−0.0023 (11)	−0.0005 (11)
C6	0.0288 (17)	0.0228 (14)	0.0234 (13)	−0.0047 (12)	0.0053 (11)	0.0019 (11)
C7	0.0453 (19)	0.0306 (15)	0.056 (2)	−0.0046 (14)	−0.0128 (18)	0.0135 (16)
C8	0.0299 (17)	0.0339 (16)	0.070 (2)	−0.0022 (13)	0.0128 (18)	0.0021 (16)
C9	0.070 (2)	0.0327 (17)	0.0347 (17)	−0.0212 (17)	0.0122 (17)	−0.0068 (15)

### Geometric parameters (Å, º)

**Table:**

S1—C4	1.718 (3)	C4—H4	0.9500
S1—C1	1.737 (3)	C6—C7	1.507 (4)
O1—C5	1.216 (3)	C6—C8	1.511 (4)
O2—C5	1.337 (3)	C6—C9	1.512 (4)
O2—C6	1.484 (3)	C7—H7A	0.9800
N1—C5	1.356 (3)	C7—H7B	0.9800
N1—C1	1.396 (3)	C7—H7C	0.9800
N1—H1N	0.90 (2)	C8—H8A	0.9800
C1—C2	1.365 (4)	C8—H8B	0.9800
C2—C3	1.418 (4)	C8—H8C	0.9800
C2—H2	0.9500	C9—H9A	0.9800
C3—C4	1.350 (4)	C9—H9B	0.9800
C3—H3	0.9500	C9—H9C	0.9800
			
C4—S1—C1	90.88 (14)	C7—C6—C8	112.6 (3)
C5—O2—C6	121.3 (2)	O2—C6—C9	103.0 (2)
C5—N1—C1	124.9 (2)	C7—C6—C9	110.2 (3)
C5—N1—H1N	117 (2)	C8—C6—C9	111.0 (3)
C1—N1—H1N	118 (2)	C6—C7—H7A	109.5
C2—C1—N1	125.4 (2)	C6—C7—H7B	109.5
C2—C1—S1	111.5 (2)	H7A—C7—H7B	109.5
N1—C1—S1	122.77 (19)	C6—C7—H7C	109.5
C1—C2—C3	112.2 (3)	H7A—C7—H7C	109.5
C1—C2—H2	123.9	H7B—C7—H7C	109.5
C3—C2—H2	123.9	C6—C8—H8A	109.5
C4—C3—C2	113.0 (3)	C6—C8—H8B	109.5
C4—C3—H3	123.5	H8A—C8—H8B	109.5
C2—C3—H3	123.5	C6—C8—H8C	109.5
C3—C4—S1	112.3 (2)	H8A—C8—H8C	109.5
C3—C4—H4	123.8	H8B—C8—H8C	109.5
S1—C4—H4	123.8	C6—C9—H9A	109.5
O1—C5—O2	126.4 (2)	C6—C9—H9B	109.5
O1—C5—N1	123.9 (2)	H9A—C9—H9B	109.5
O2—C5—N1	109.6 (2)	C6—C9—H9C	109.5
O2—C6—C7	110.9 (2)	H9A—C9—H9C	109.5
O2—C6—C8	108.8 (2)	H9B—C9—H9C	109.5
			
C5—N1—C1—C2	177.5 (3)	C1—S1—C4—C3	−0.9 (3)
C5—N1—C1—S1	−8.9 (4)	C6—O2—C5—O1	3.3 (4)
C4—S1—C1—C2	0.9 (2)	C6—O2—C5—N1	−176.4 (2)
C4—S1—C1—N1	−173.5 (2)	C1—N1—C5—O1	−7.8 (4)
N1—C1—C2—C3	173.6 (2)	C1—N1—C5—O2	171.9 (2)
S1—C1—C2—C3	−0.7 (3)	C5—O2—C6—C7	54.5 (3)
C1—C2—C3—C4	0.0 (4)	C5—O2—C6—C8	−69.8 (3)
C2—C3—C4—S1	0.7 (4)	C5—O2—C6—C9	172.4 (3)

### Hydrogen-bond geometry (Å, º)

**Table:**

*D*—H···*A*	*D*—H	H···*A*	*D*···*A*	*D*—H···*A*
N1—H1*N*···O1^i^	0.90 (2)	2.04 (2)	2.920 (3)	165 (3)
C7—H7*A*···O1	0.98	2.33	2.938 (4)	119
C8—H8*C*···O1	0.98	2.55	3.109 (4)	116

Symmetry code: (i) −*x*+1/2, *y*, *z*+1/2.
